# 

**Published:** 1876-06

**Authors:** 


### Table of Contents.

### ORIGINAL COMMUNICATIONS.

Remarks on Inflammation and its Curability........................ 129
Oxygen Gas a Remedy in Disease........................ 133
Case of Tænia Solium Similating Pleuritis........................ 152
Child Crying in Utero........................

### ATLANTA ACADEMY OF MEDICINE.

Cherry Seed lodged in Posterior Nares—Ham Poisoning - Impotence Amputation at Ankle Joint—Eneucleation of Eye-ball —Partial Deafness—Excessive Irritation of Urethra—Epileptic Fits—Typhoid Fever—Report of case of Tumor—Uterine Hemorrhage--Hereditary Tendency of Disease........................ 153
Clinic Atlanta Medical College........................ 167

### OUR EXCHANGES.

Intestinal Obstruction........................ 175
Diminution of Spleen under the Hypodermic Use of Ergot........................ 177
New York Academy of Medicine........................ 178
Diseases of the Viscera........................ 180
Nervous Aphonia Cured by Inhalation of Chloroform........................
Ergot in the Treatment of Glactorrhœa and Mastitis........................ 181
Circulatory Disturbances in Traumatic Lesions of the Brain........................
"No More Ovariotomy"........................ 182
Caustics in the Treatment of Diseases of the Neck of the Bladder........................ 183
Esmarch's Method Simplified........................
Damiana........................
Muscular Exertion as a Cause of Albuminuria........................ 184
Pruritus........................

### BIBLIOGRAPHICAL.

Catalogue of National Medical Library........................ 184
Pamphlets Received........................ 185

### EDITORIAL

An Apology........................ 186

European Correspondence........................

The Convention of Medical Colleges........................ 187

International Medical College........................ 190

The Michigan University and Homœopathy........................

Impudence........................ 191

Transactions of State Association........................ 192

Correction........................

